# The Gut-Lung Axis in Health and Respiratory Diseases: A Place for Inter-Organ and Inter-Kingdom Crosstalks

**DOI:** 10.3389/fcimb.2020.00009

**Published:** 2020-02-19

**Authors:** Raphaël Enaud, Renaud Prevel, Eleonora Ciarlo, Fabien Beaufils, Gregoire Wieërs, Benoit Guery, Laurence Delhaes

**Affiliations:** ^1^CHU de Bordeaux, CRCM Pédiatrique, CIC 1401, Bordeaux, France; ^2^Univ. Bordeaux, Centre de Recherche Cardio-Thoracique de Bordeaux, U1045, Bordeaux, France; ^3^CHU de Bordeaux, Univ. Bordeaux, FHU ACRONIM, Bordeaux, France; ^4^CHU de Bordeaux, Médecine Intensive Réanimation, Bordeaux, France; ^5^Infectious Diseases Service, Department of Medicine, Lausanne University Hospital and University of Lausanne, Lausanne, Switzerland; ^6^CHU de Bordeaux, Service d'Explorations Fonctionnelles Respiratoires, Bordeaux, France; ^7^Clinique Saint Pierre, Department of Internal Medicine, Ottignies, Belgium; ^8^CHU de Bordeaux: Laboratoire de Parasitologie-Mycologie, Univ. Bordeaux, Bordeaux, France

**Keywords:** Gut-Lung Axis, Mycobiota, Microbiome, Respiratory disease, Dysbiosis

## Abstract

The gut and lungs are anatomically distinct, but potential anatomic communications and complex pathways involving their respective microbiota have reinforced the existence of a gut–lung axis (GLA). Compared to the better-studied gut microbiota, the lung microbiota, only considered in recent years, represents a more discreet part of the whole microbiota associated to human hosts. While the vast majority of studies focused on the bacterial component of the microbiota in healthy and pathological conditions, recent works have highlighted the contribution of fungal and viral kingdoms at both digestive and respiratory levels. Moreover, growing evidence indicates the key role of inter-kingdom crosstalks in maintaining host homeostasis and in disease evolution. In fact, the recently emerged GLA concept involves host–microbe as well as microbe–microbe interactions, based both on localized and long-reaching effects. GLA can shape immune responses and interfere with the course of respiratory diseases. In this review, we aim to analyze how the lung and gut microbiota influence each other and may impact on respiratory diseases. Due to the limited knowledge on the human virobiota, we focused on gut and lung bacteriobiota and mycobiota, with a specific attention on inter-kingdom microbial crosstalks which are able to shape local or long-reached host responses within the GLA.

## Introduction

Recent advances in microbiota explorations have led to an improved knowledge of the communities of commensal microorganisms within the human body. Human skin and mucosal surfaces are associated with rich and complex ecosystems (microbiota) composed of bacteria (bacteriobiota), fungi (mycobiota), viruses (virobiota), phages, archaea, protists, and helminths (Cho and Blaser, 2012). The role of the gut bacteriobiota in local health homeostasis and diseases is being increasingly investigated, but its long-distance impacts still need to be clarified (Chiu et al., 2017). Among the relevant inter-organ connections, the gut–lung axis (GLA) remains less studied than the gut–brain axis. So far, microbiota studies mainly focused on the bacterial component, neglecting other microbial kingdoms. However, the understanding of mycobiota involvement in human health and inter-organ connections should not be overlooked (Nguyen et al., 2015; Enaud et al., 2018). Viruses are also known to be key players in numerous respiratory diseases and to interact with the human immune system, but technical issues still limit the amount of data regarding virobiota (Mitchell and Glanville, 2018). Therefore, we will focus on bacterial and fungal components of the microbiota and their close interactions that are able to shape local or long-reached host responses within the GLA. While GLA mycobiota also influences chronic gut diseases such as IBD, we will not address this key role in the present review: we aimed at analyzing how lung and gut bacteriobiota and mycobiota influence each other, how they interact with the human immune system, and their role in respiratory diseases.

## Microbial Interactions Within the Gut–Lung Axis

### Inter-Kingdom Crosstalk Within the Gut Microbiota

The gut microbiota has been the most extensively investigated. The majority of genes (99%) amplified in human stools are from bacteria, which are as numerous as human cells and comprise 150 distinct bacterial species, belonging mainly to Firmicutes and Bacteroidetes phyla. Proteobacteria, Actinobacteria, Cyanobacteria, and Fusobacteria are also represented in healthy people (Sekirov et al., 2010; Human Microbiome Project Consortium, 2012).

More recently, fungi have been recognized as an integral part of our commensal flora, and their role in health and diseases is increasingly considered (Huffnagle and Noverr, 2013; Huseyin et al., 2017). Fungi are about 100 times larger than bacteria, so even if fungal sequences are 100 to 1,000 times less frequent than bacterial sequences, fungi must not be neglected in the gastrointestinal ecosystem. In contrast with the bacteriobiota, the diversity of the gut mycobiota in healthy subjects is limited to few genera, with a high prevalence of *Saccharomyces cerevisiae, Malassezia restricta*, and *Candida albicans* (Nash et al., 2017).

Although often dichotomized due to technical and analysis sequencing issues, critical interactions exist between bacteriobiota and mycobiota (Peleg et al., 2010). The most appropriate approach to decipher the role of gut microbiota is therefore considering the gut as an ecosystem in which inter-kingdom interactions occur and have major implications as suggested by the significant correlations between the gut bacteriobiota and mycobiota profiles among healthy subjects (Hoffmann et al., 2013). Yeasts, e.g., *Saccharomyces boulardii* and *C. albicans*, or fungus wall components, e.g., β-glucans, are able to inhibit the growth of some intestinal pathogens (Zhou et al., 2013; Markey et al., 2018). *S. boulardii* also produces proteases or phosphatases that inactivate the toxins produced by intestinal bacteria such as *Clostridium difficile* and *Escherichia coli* (Castagliuolo et al., 1999; Buts et al., 2006). In addition, at physiological state and during gut microbiota disturbances (e.g., after a course of antibiotics), fungal species may take over the bacterial functions of immune modulation, preventing mucosal tissue damages (Jiang et al., 2017). *Vice versa*, bacteria can also modulate fungi: fatty acids locally produced by bacteria impact on the phenotype of *C. albicans* (Noverr and Huffnagle, 2004; Tso et al., 2018).

Beside the widely studied gut microbiota, microbiotas of other sites, including the lungs, are essential for host homeostasis and disease. The lung microbiota is now recognized as a cornerstone in the physiopathology of numerous respiratory diseases (Soret et al., 2019; Vandenborght et al., 2019).

### Inter-Kingdom Crosstalk Within the Lung Microbiota

The lung microbiota represents a significantly lower biomass than the gut microbiota: about 10 to 100 bacteria per 1,000 human cells (Sze et al., 2012). Its composition depends on the microbial colonization from the oropharynx and upper respiratory tract through salivary micro-inhalations, on the host elimination abilities (especially coughing and mucociliary clearance), on interactions with the host immune system, and on local conditions for microbial proliferation, such as pH or oxygen concentration (Gleeson et al., 1997; Wilson and Hamilos, 2014). The predominant bacterial phyla in lungs are the same as in gut, mainly Firmicutes and Bacteroidetes followed by Proteobacteria and Actinobacteria (Charlson et al., 2011). In healthy subjects, the main identified fungi are usually environmental: Ascomycota (*Aspergillus, Cladosporium, Eremothecium*, and *Vanderwaltozyma)* and Microsporidia (*Systenostrema*) (Nguyen et al., 2015; Vandenborght et al., 2019). In contrast to the intestinal or oral microbiota, data highlighting the interactions between bacteria and fungi in the human respiratory tract are more scattered (Delhaes et al., 2012; Soret et al., 2019). However, data from both *in vitro* and *in vivo* studies suggest relevant inter-kingdom crosstalk (Delhaes et al., 2012; Xu and Dongari-Bagtzoglou, 2015; Lof et al., 2017; Soret et al., 2019). This dialogue may involve several pathways as physical interaction, quorum-sensing molecules, production of antimicrobial agents, immune response modulation, and nutrient exchange (Peleg et al., 2010). Synergistic interactions have been documented between *Candida* and *Streptococcus*, such as stimulation of *Streptococcus* growth by *Candida*, increasing biofilm formation, or enhancement of the *Candida* pathogenicity by *Streptococcus* (Diaz et al., 2012; Xu et al., 2014). *In vitro* studies exhibited an increased growth of *Aspergillus fumigatus* in presence of *Pseudomonas aeruginosa*, due to the mold's ability in to assimilate *P. aeruginosa*-derived volatile sulfur compounds (Briard et al., 2019; Scott et al., 2019). However, the lung microbiota modulation is not limited to local inter-kingdom crosstalk and also depends on inter-compartment crosstalk between the gut and lungs.

### Microbial Inter-compartment Crosstalk

From birth throughout the entire life span, a close correlation between the composition of the gut and lung microbiota exists, suggesting a host-wide network (Grier et al., 2018). For instance, modification of newborns' diet influences the composition of their lung microbiota, and fecal transplantation in rats induces changes in the lung microbiota (Madan et al., 2012; Liu et al., 2017).

The host's health condition can impact this gut–lung interaction too. In cystic fibrosis (CF) newborns, gut colonizations with *Roseburia, Dorea, Coprococcus, Blautia*, or *Escherichia* presaged their respiratory appearance, and their gut and lung abundances are highly correlated over time (Madan et al., 2012). Similarly, the lung microbiota is enriched with gut bacteria, such as *Bacteroides* spp., after sepsis (Dickson et al., 2016).

Conversely, lung microbiota may affect the gut microbiota composition. In a pre-clinical model, influenza infection triggers an increased proportion of *Enterobacteriaceae* and decreased abundances of *Lactobacilli* and *Lactococci* in the gut (Looft and Allen, 2012). Consistently, lipopolysaccharide (LPS) instillation in the lungs of mice is associated with gut microbiota disturbances (Sze et al., 2014).

Although gastroesophageal content inhalations and sputum swallowing partially explain this inter-organ connection, GLA also involves indirect communications such as host immune modulation.

## Gut–Lung Axis Interactions With the Human Immune System

### Gut Microbiota and Local Immunity

Gut microbiota effects on the local immune system have been extensively reviewed (Elson and Alexander, 2015). Briefly, the gut microbiota closely interacts with the mucosal immune system using both pro-inflammatory and regulatory signals (Skelly et al., 2019). It also influences neutrophil responses, modulating their ability to extravasate from blood (Karmarkar and Rock, 2013). Toll-like receptor (TLR) signaling is essential for microbiota-driven myelopoiesis and exerts a neonatal selection shaping the gut microbiota with long-term consequences (Balmer et al., 2014; Fulde et al., 2018). Moreover, the gut microbiota communicates with and influences immune cells expressing TLR or GPR41/43 by means of microbial associated molecular patterns (MAMPs) or short-chain fatty acids (SCFAs) (Le Poul et al., 2003). Data focused on the gut mycobiota's impact on the immune system are sparser. Commensal fungi seem to reinforce bacterial protective benefits on both local and systemic immunity, with a specific role for mannans, a highly conserved fungal wall component. Moreover, fungi are able to produce SCFAs (Baltierra-Trejo et al., 2015; Xiros et al., 2019). Therefore, gut mycobiota perturbations could be as deleterious as bacteriobiota ones (Wheeler et al., 2016; Jiang et al., 2017).

### Lung Microbiota and Local Immunity

A crucial role of lung microbiota in the maturation and homeostasis of lung immunity has emerged over the last few years (Dickson et al., 2018). Colonization of the respiratory tract provides essential signals for maturing local immune cells with long-term consequences (Gollwitzer et al., 2014). Pre-clinical studies confirm the causality between airway microbial colonization and the regulation and maturation of the airways' immune cells. Germ-free mice exhibit increased local Th2-associated cytokine and IgE production, promoting allergic airway inflammation (Herbst et al., 2011). Consistently, lung exposure to commensal bacteria reduces Th2-associated cytokine production after an allergen challenge and induces regulatory cells early in life (Russell et al., 2012; Gollwitzer et al., 2014). The establishment of resident memory B cells in lungs also requires encountering lung microbiota local antigens, especially regarding immunity against viruses such as influenza (Allie et al., 2019).

Interactions between lung microbiota and immunity are also a two-way process; a major inflammation in the lungs can morbidly transform the lung microbiota composition (Molyneaux et al., 2013).

### Long-Reaching Immune Modulation Within the Gut–Lung Axis

Beyond the local immune regulation by the site-specific microbiota, the long-reaching immune impact of gut microbiota is now being recognized, especially on the pulmonary immune system (Chiu et al., 2017). The mesenteric lymphatic system is an essential pathway between the lungs and the intestine, through which intact bacteria, their fragments, or metabolites (e.g., SCFAs) may translocate across the intestinal barrier, reach the systemic circulation, and modulate the lung immune response (Trompette et al., 2014; Bingula et al., 2017; McAleer and Kolls, 2018). SCFAs, mainly produced by the bacterial dietary fibers' fermentation especially in case of a high-fiber diet (HFD), act in the lungs as signaling molecules on resident antigen-presenting cells to attenuate the inflammatory and allergic responses (Anand and Mande, 2018; Cait et al., 2018). SCFA receptor–deficient mice show increased inflammatory responses in experimental models of asthma (Trompette et al., 2014). Fungi, including *A. fumigatus*, can also produce SCFAs or create a biofilm enhancing the bacterial production of SCFAs, but on the other hand, bacterial SCFAs can dampen fungal growth (Hynes et al., 2008; Baltierra-Trejo et al., 2015; Xiros et al., 2019). The impact of fungal production of SCFAs on the host has not been assessed so far.

Other important players of this long-reaching immune effect are gut segmented filamentous bacteria (SFBs), a commensal bacteria colonizing the ileum of most animals, including humans, and involved in the modulation of the immune system's development (Yin et al., 2013). SFBs regulate CD4+ T-cell polarization into the Th17 pathway, which is implicated in the response to pulmonary fungal infections and lung autoimmune manifestations (McAleer et al., 2016; Bradley et al., 2017). Recently, innate lymphoid cells, involved in tissue repair, have been shown to be recruited from the gut to the lungs in response to inflammatory signals upon IL-25 (Huang et al., 2018). Finally, intestinal TLR activation, required for the NF-κB–dependent pathways of innate immunity and inflammation, is associated with an increased influenza-related lung response in mice (Ichinohe et al., 2011).

Other mechanisms may be involved in modulating the long-reaching immune response related to gut microbiota, exemplified by the increased number of mononuclear leukocytes and an increased phagocytic and lytic activity after treatment with *Bifidobacterium lactis* HN019 probiotics (Gill et al., 2001). Diet, especially fiber intake, which increases the systemic level of SCFAs, or probiotics influence the pulmonary immune response and thus impact the progression of respiratory disorders (King et al., 2007; Varraso et al., 2015; Anand and Mande, 2018).

The GLA immune dialogue remains a two-way process. For instance, *Salmonella* nasal inoculation promotes a *Salmonella*-specific gut immunization which depends on lung dendritic cells (Ruane et al., 2013). Respiratory influenza infection also modulates the composition of the gut microbiota as stated above. These intestinal microbial disruptions seem to be unrelated to an intestinal tropism of influenza virus but mediated by Th17 cells (Wang et al., 2014).

In summary, GLA results from complex interactions between the different microbial components of both the gut and lung microbiotas combined with local and long-reaching immune effects. All these interactions strongly suggest a major role for the GLA in respiratory diseases, as recently documented in a mice model (Skalski et al., 2018).

## Gut–Lung Axis in Respiratory Diseases

### Acute Infectious Diseases

Regarding influenza infection and the impact of gut and lung microbiota, our knowledge is still fragmentary; human data are not yet available. However, antibiotic treatment causes significantly reduced immune responses against influenza virus in mice (Ichinohe et al., 2011). Conversely, influenza-infected HFD-fed mice exhibit increased survival rates compared to infected controls thanks to an enhanced generation of Ly6c-patrolling monocytes. These monocytes increase the numbers of macrophages that have a limited capacity to produce CXCL1 locally, reducing neutrophil recruitment to the airways and thus tissue damage. In parallel, diet-derived SCFAs boost CD8+ T-cell effector function in HFD-fed mice (Trompette et al., 2018).

Both lung and gut microbiota are essential against bacterial pneumonia. The lung microbiota is able to protect against respiratory infections with *Streptococcus pneumoniae* and *Klebsiella pneumonia*e by priming the pulmonary production of granulocyte-macrophage colony-stimulating factor (GM-CSF) via IL-17 and Nod2 stimulation (Brown et al., 2017). The gut microbiota also plays a crucial role in response to lung bacterial infections. Studies on germ-free mice showed an increased morbidity and mortality during *K. pneumoniae, S. pneumoniae*, or *P. aeruginosa* acute lung infection (Fagundes et al., 2012; Fox et al., 2012; Brown et al., 2017). The use of broad-spectrum antibiotic treatments, to disrupt mouse gut microbiota, results in worse outcome in lung infection mouse models (Schuijt et al., 2016; Robak et al., 2018). Mechanistically, alveolar macrophages from mice deprived of gut microbiota through antibiotic treatment are less responsive to stimulation and show reduced phagocytic capacity (Schuijt et al., 2016). Interestingly, priming of antibiotic-treated animals with TLR agonists restores resistance to pulmonary infections (Fagundes et al., 2012). SFBs appear to be an important gut microbiota component for lung defense against bacterial infection thanks to their capacity to induce the production of the Th17 cytokine, IL-22, and to increase neutrophil counts in the lungs during *Staphylococcus aureus* pneumonia (Gauguet et al., 2015).

Modulating chronic infectious diseases will similarly depend on gut and lung microbiotas. For instance, *Mycobacterium tuberculosis* infection severity is correlated with gut microbiota (Namasivayam et al., 2018).

### Chronic Respiratory Diseases

Multiple studies have addressed the impact of gut and lung microbiota on chronic respiratory diseases such as chronic obstructive pulmonary disease (COPD), asthma, and CF (Table 1).

**Table 1:** Gut–lung axis in human chronic respiratory diseases.

**Lung disease**	**Microbiota disorders in**		**Comments**	**References**
	**Lungs**	**Gut**		
Chronic obstructive pulmonary disease (COPD)	Decreased lung microbiota diversity, *Proteobacteria* expansion		Associated with both COPD severity and exacerbations	Garcia-Nuñez et al., 2014; Wang et al., 2016; Mayhew et al., 2018
		Increased gastro-intestinal permeability and level of circulating gut microbiota-dependent trimethylamine-N-oxide	Associated with long-term all-cause mortality in COPD patients	Ottiger et al., 2018
Asthma	Proteobacteria (*Haemophilus, Neisseria, Pseudomonas, Rickettsia* and *Moraxella* species) and Firmicutes (*Lactobacillus*)		Overrepresented in asthmatic patients and/or associated with an uncontrolled asthma	Hilty et al., 2010; Marri et al., 2013; Huang et al., 2015; Denner et al., 2016; Zhang et al., 2016; Durack et al., 2017; Sverrild et al., 2017
	Bacteroidetes and Fusobacteria *Moraxella catarrhalis, Bacteroides, Haemophilus* and *Streptococcus*		Reduced in asthmatic patients Associated with worse FEV_1_ and higher sputum neutrophil counts	Zhang et al., 2016 Green et al., 2014; Sverrild et al., 2017
	*Malassezia*		Overrepresented in asthmatic patients	van Woerden et al., 2013
	*Aspergillus fumigatus*		Associated with corticosteroid treatment	Fraczek et al., 2018
		Early-life perturbations - Low gut microbial diversity - Increased bacterial abundance of *Clostridium, Streptococcus* and *Bacteroides fragilis* - Decreased bacterial abundances of *Lachnospira, Veillonella, Faecalibacterium, Rothia Bacteroides* and *Bifidobacterium* - Increased fungal abundances of *Saccharomyces* and *Pichia kudriavzevii* - Decreased fungal abundances of *Candida tropicalis* and *Debaryomyces hansenii*	Increased risk of childhood asthma development	Björkstén et al., 2001; Penders et al., 2007; Vael et al., 2008; van Nimwegen et al., 2011; Abrahamsson et al., 2012; Arrieta et al., 2015, 2018; Stiemsma et al., 2016
Cystic fibrosis (CF)	Decreased lung microbiota diversity		Correlated with the disease evolution and associated with exacerbation	Zhao et al., 2012; Stokell et al., 2015; Frayman et al., 2017
	*Streptococcus, Prevotella, Rothia, Veillonella, Acintomyces, Neisseria, Haemophilus, Gemella*		Major bacterial taxa in CF pediatric patients	Worlitzsch et al., 2009; Madan et al., 2012; Renwick et al., 2014; Coburn et al., 2015; Frayman et al., 2017
	*Streptococcus,Prevotella, Rothia, Veillonella,Acintomyces, Pseudomonas*		Major bacterial taxa in CF adult patients	Cox et al., 2010; Hampton et al., 2014; Coburn et al., 2015; Frayman et al., 2017
	*Candida albicans and Aspergillus fumigatus*		Major fungi isolated in CF patient; *C. albicans* was more likely co-associated with *P. aeruginosa*	Delhaes et al., 2012; Willger et al., 2014; Kim et al., 2015; Nguyen et al., 2016
		Decrease of *Parabacteroides*	Predictive of an airway colonization with *Pseudomonas aeruginosa* Association	Hoen et al., 2015
		Increased abundances of *Staphylococcus, Streptococcus, Escherichia coli* and *Veillonella dispar*	Association with CF intestinal inflammation	Hoffman et al., 2014; Enaud et al., 2019 Enaud et al., 2019
		Decreased abundances of *Bacteroides, Bifidobacterium adolescentis* and *Faecalibacterium prausnitzii*	Association with with CF intestinal inflammation	

Decreased lung microbiota diversity and *Proteobacteria* expansion are associated with both COPD severity and exacerbations (Garcia-Nuñez et al., 2014; Wang et al., 2016, 2018; Mayhew et al., 2018). The fact that patients with genetic mannose binding lectin deficiency exhibit a more diverse pulmonary microbiota and a lower risk of exacerbation suggests not only association but also causality (Dicker et al., 2018). Besides the lung flora, the gut microbiota is involved in exacerbations, as suggested by the increased gastrointestinal permeability in patients admitted for COPD exacerbations (Sprooten et al., 2018). Whatever the permeability's origin (hypoxemia or pro-inflammatory status), the level of circulating gut microbiota–dependent trimethylamine-N-oxide has been associated with mortality in COPD patients (Ottiger et al., 2018). This association being explained by comorbidities and age, its impact *per se* is not guaranteed. Further studies are warranted to investigate the role of GLA in COPD and to assess causality.

Early-life perturbations in fungal and bacterial gut colonization, such as low gut microbial diversity, e.g., after neonatal antibiotic use, are critical to induce childhood asthma development (Abrahamsson et al., 2014; Metsälä et al., 2015; Arrieta et al., 2018). This microbial disruption is associated with modifications of fecal SCFA levels (Arrieta et al., 2018). Causality has been assessed in murine models. Inoculation of the bacteria absent in the microbiota of asthmatic patients decreases airways inflammation (Arrieta et al., 2015). Furthermore, *Bacteroides fragilis* seems to play a major role in immune homeostasis, balancing the host systemic Th1/Th2 ratio and therefore conferring protection against allergen-induced airway disorders (Mazmanian et al., 2005; Panzer and Lynch, 2015; Arrieta et al., 2018). Nevertheless, it is still not fully deciphered, as some studies conversely found that an early colonization with *Bacteroides*, including *B. fragilis*, could be an early indicator of asthma later in life (Vael et al., 2008). Regarding fungi, gut fungal overgrowth (after antibiotic administration or a gut colonization protocol with *Candida* or *Wallemia mellicola*) increases the occurrence of asthma via IL-13 without any fungal expansion in the lungs (Noverr et al., 2005; Wheeler et al., 2016; Skalski et al., 2018). The prostaglandin E2 produced in the gut by *Candida* can reach the lungs and promotes lung M2 macrophage polarization and allergic airway inflammation (Kim et al., 2014). In mice, a gut overrepresentation of *W. mellicola* associated with several intestinal microbiome disturbances appears to have long-reaching effects on the pulmonary immune response and severity of asthma, by involving the Th2 pathways, especially IL-13 and to a lesser degree IL-17, goblet cell differentiation, fibroblasts activation, and IgE production by B cells (Skalski et al., 2018). Taken together, these results indicate that the GLA, mainly through the gut microbiota, is likely to play a major role in asthma.

In CF patients, gut and lung microbiota are distinct from those of healthy subjects, and disease progression is associated with microbiota alterations (Madan et al., 2012; Stokell et al., 2015; Nielsen et al., 2016). Moreover, the bacterial abundances at both sites are highly correlated and have similar trends over time (Madan et al., 2012), especially regarding *Streptococcus*, which is found in higher proportion in CF stools, gastric contents, and sputa (Al-Momani et al., 2016; Nielsen et al., 2016). Moreover, CF patients with a documented intestinal inflammation exhibit a higher *Streptococcus* abundance in the gut (Enaud et al., 2019), suggesting the GLA's involvement in intestinal inflammation. Of note, gut but not lung microbiota alteration is associated with early-life exacerbations: some gut microbiota perturbations, such as a decrease of *Parabacteroides*, are predictive of airway colonization with *P. aeruginosa* (Hoen et al., 2015). Furthermore, oral administration of probiotics to CF patients leads to a decreased number of exacerbations (Anderson et al., 2016). While the mycobiota has been recently studied in CF (Nguyen et al., 2015; Soret et al., 2019), no data on the role of the fungal component of the GLA are currently available in CF, which deserves to be more widely studied.

The role of inter-compartment and inter-kingdom interactions within the GLA in those pulmonary diseases now has to be further confirmed and causality to be assessed. Diet, probiotics, or more specific modulations could be, in the near future, novel essential tools in therapeutic management of these respiratory diseases.

## Conclusion

**Figure 1:**
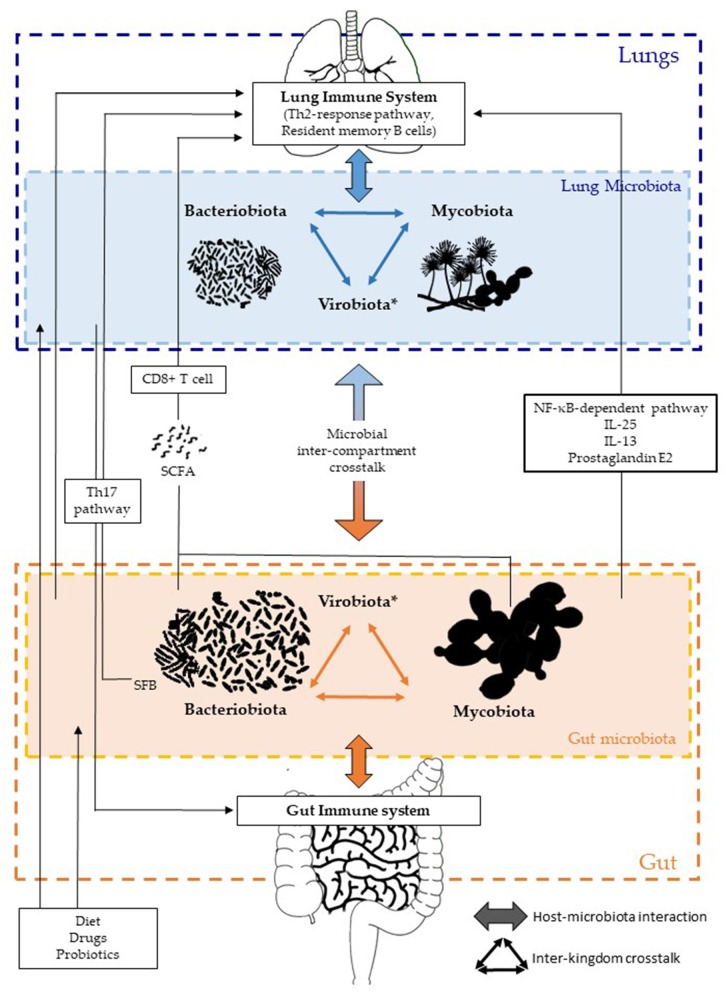
Inter-kingdom and inter-compartment crosstalks within the gut–lung axis. Bacteriobiota, mycobiota, and virobiota are closely interacting within each organ by either direct or indirect mechanisms shaping each other. Gut microbiota influences both the gut immune system and the lung immune system via local or long-reaching interactions, which involve either CD8+ T cell, Th17, IL-25, IL-13, prostaglandin E2, and/or NF-κB–dependent pathways. The lung microbiota impacts the mucosal immunity and contributes to immune tolerance, through neutrophil recruitment, production of pro-inflammatory cytokines mediated by receptor 2 (TLR2), and the release of antimicrobial peptides, such as β-defensin 2 stimulated by T helper 17 (Th17) cells. On the other hand, the lung microbiota also influences the gut immune system, but precise mechanisms remain to be deciphered, even if an intestinal microbial disruption has been associated with Th17 cell mediation after influenza virus lung infection. Several factors are well-known to influence the composition of the intestinal and/or lung microbiota, such as diet, drugs, and probiotics. *Of note, the virobiota is not covered in this review.

The gut–lung axis or GLA has emerged as a specific axis with intensive dialogues between the gut and lungs, involving each compartment in a two-way manner, with both microbial and immune interactions (Figure 1). Each kingdom and compartment plays a crucial role in this dialogue, and consequently in host health and diseases. The roles of fungal and viral kingdoms within the GLA still remain to be further investigated. Their manipulation, as for the bacterial component, could pave the way for new approaches in the management of several respiratory diseases such as acute infections, COPD, asthma, and/or CF.

## Author Contributions

RE, RP, EC, FB, GW, BG, and LD conceptualized and wrote the manuscript.

### Conflict of Interest

The authors declare that the research was conducted in the absence of any commercial or financial relationships that could be construed as a potential conflict of interest.
